# Attraction to equilibria in discrete population models with delayed feedbacks: stage-structure versus age-structure

**DOI:** 10.1007/s00285-025-02200-5

**Published:** 2025-03-20

**Authors:** Hassan A. El-Morshedy, Alfonso Ruiz-Herrera

**Affiliations:** 1 Departament of Mathematics, Faculty of Science, Damietta University, Damietta, Egypt; 2 Departamento de Matemáticas, University of Oviedo, Oviedo, Spain

**Keywords:** Stage structure, Time delays, Attraction, Clark’s model, Reproduction strategy, Development rate, 37C70, 37N35

## Abstract

Time delays and stage structure are common features of most biological populations. This paper aims to describe the influence of these features through simple models. Regarding the role of time delays, we discuss the dynamical differences between populations where the main intraspecific competition episodes occur during the reproduction period or during a different one. The conclusion is that the second situation is generally more prone to generate long-term oscillations. Regarding the role of the stage structure, we show that the shape of the adult recruitment plays a key role. Particularly, adult recruitments associated with contest-type intraspecific competitions do not produce long-term oscillations. From a mathematical point of view, we offer two general criteria of global attraction in discrete systems valid for non-monotone models.

## Introduction

The life cycles of plants and animals show great diversity of ecological patterns (Mjølhus et al. 2005; Neubert and Caswell 2000; Wikan 2012). Some individuals live for a few hours and others can live for centuries. Specialized stages may exist for dispersal or latency. In addition, metamorphosis can take the same individual through totally different life stages during its lifetime. The model

1.1 $$\begin{aligned} x_{n+1}=h(x_{n}),\;\;\;\;\;\;n=0,1,... \end{aligned}$$

with $h:[0,+\infty )\longrightarrow [0,+\infty )$ a continuous function does not always capture this diversity of ecological patterns. For example, (1.1) neglects some variables such as age structure, reproduction strategy, maturation periods, or hatching times (Elaydi 2005; Liz and Ruiz-Herrera 2012a). Introducing these variables in (1.1) implies increasing the dimension of the phase space of the model. Typically, stage structure involves considering a system of difference equations and maturation or hatching times imply the use of time delays. Describing how the dynamical behavior of models changes when stage structure or time delays are introduced is far from a trivial task. We stress that model (1.1) can already exhibit a broad range of dynamical behaviors including the attraction to an equilibrium or a periodic point, and chaotic dynamics, (Elaydi 2005; Liz and Ruiz-Herrera 2012a).

A natural problem in ecology is to analyze when a population follows simple dynamical behaviors. Nevertheless, the vital rates, e.g., survival rates, development, and reproduction, almost always depend on the population density and/or developmental stage, leading to nonlinear models. In these cases, an exponential growth rate does not determine the dynamic behavior of these models, but rather a subtle and non-systematic analysis. The number of techniques available in the literature that guarantee the existence of a global attracting equilibrium is relatively scarce (El-Morshedy and Liz 2005; El-Morshedy and López 2008; Kocic and Ladas 1992, 1995; Singer 1978). The main two techniques are the construction of adequate Lyapunov functions and the theory of monotone systems, see Agarwal (2000), Smith (1998). However, both tools present strong limitations. The use of Lyapunov functions is normally difficult because no methodology exists to construct them and the theory of monotone systems is not applicable when the populations have unimodal birth rates.

The purpose of this paper is two-fold. First, we describe some biological insights into the dynamical consequences of the reproduction strategy, developmental rate, and delayed feedback concerning the absence of oscillations. We shall focus on several classical models and compare some relevant patterns. Second, we provide new criteria for global attraction in discrete models. Specifically, we propose a general methodology for studying scalar equations with delays that is reminiscent of an extension of the theory of monotone systems known as the “decomposing+embedding” method. Moreover, we propose an ad hoc methodology based on subtly using several properties of scalar equations to study a class of age-structured models.

The structure of the paper is as follows. Section 2 describes a general class of matrix models and introduces some basic notions on species life cycles. Section 3 studies the influence of time delays on the classical Clark model (Clark 1976). For many species, major episodes of intraspecific competition occur during the breeding period. However, some species do not have this behavior, e.g., most tick populations (El-Morshedy and Ruiz-Herrera 2024; Huang et al. 2022; Zhang and Wu 2020). We will show the differences and similarities in the dynamic behaviors of both ecological situations. Section 4 analyzes a general class of population models with two age groups, juveniles and adults. Finally, we discuss our findings, emphasizing the connections between age and stage structures.

## Simple matrix models

Stage-structured models are versatile tools capable of incorporating the diversity of biological features of most plant and animal life cycles. Neubert and Caswell (2000) proposed the modeling framework

2.1 $$\begin{aligned} \left\{ \begin{array}{lll} x_{n+1}=\mu _{1}(x_{n},y_{n}) (1-p)x_{n}+g(y_{n})y_{n}\\ y_{n+1}=\mu _{1}(x_{n},y_{n})p x_{n}+\mu _{2}(x_{n},y_{n})y_{n} \end{array}\right. \end{aligned}$$

with $x_{n}$, $y_{n}$ the number of juveniles and adults, respectively. The parameters $\mu _{1}, \mu _{2}\in [0,1]$ represent the fraction of juveniles and adults (respectively) at time *n* that survive to time $n+1$. The fraction of the surviving juveniles that become adults is $p\in [0,1]$. The function *g* denotes the number of newborns produced by an adult. The reader can consult (Mjølhus et al. 2005; Wikan 2012) and the references therein for relevant literature on model (2.1).

Following Neubert and Caswell (2000), model (2.1) allows us to describe four important classes of life histories depending on the reproduction strategy and developmental rate. The reproduction strategy can be classified as semelparous (reproducing only once) or iteroparous (reproducing repeatedly). Semelparity occurs when $\mu _{2}\approx 0$ and iteroparity when $\mu _{2}\in (0,1)$. Development can be classified as precocious (rapid development to maturity) or delayed. Precocious development is associated with $p\approx 1$ and delayed development with $p\in (0,1)$.

A simple formulation of a population model that involves age-structure and density-dependent recruitment is

2.2 $$\begin{aligned} x_{n+1}=\mu x_{n}+f(x_{n}) \end{aligned}$$

where $\mu \in (0,1)$ is the adult’s probability of surviving one year including the reproductive season (Clark 1976; Thieme 2018). In this model, the function $f:[0,+\infty )\longrightarrow [0,+\infty )$ represents the relationship between the current density of adults and the number of offspring that will become adults. Thus, *f* can be written as *xA*(*x*)*B*(*x*) with *A*(*x*) the birth rate and *B*(*x*) the survival probability of the offspring from time *n* to $n+1$, (Yu and Li 2022). Equation (2.2) has been extensively used as a suitable model for understanding the population dynamics of several species including whales, bobwhite quails, mosquitoes, etc, (see Brauer and Castillo-Chavez 2012, Clark 1976, Milton and Bélair 1990, Thieme 2018, Yu and Li 2022 and the references therein). We mention that (2.2) has also been employed to study the production of red blood cells (erythrocytes) in mammals (Lasota 1977). A possible limitation of (2.2) is the absence of time delays. When a model describes the dynamics of a population, time delays naturally arise from the maturation time, i.e., the recruitment to the breeding population occurs after *k* periods (Brauer and Castillo-Chavez 2012; Thieme 2018). Moreover, the survival and birth rates generally depend on a previous intraspecific competition of individuals (Brauer and Castillo-Chavez 2012; Thieme 2018). A primary extension of model (2.2) that solves these limitations could be

2.3 $$\begin{aligned} x_{n+1}=\mu x_{n}+B(x_{n-k})x_{n-k}A(x_{n-k}),\;\;\;n=0,1,2,.... \end{aligned}$$

with $k\ge 0$. It is worth mentioning that (2.3) for $k=1$ can be derived from a simple Leslie model of the form

2.4 $$\begin{aligned} \left\{ \begin{array}{lll} x_{n+1}=\alpha _{1}x_{n}+\frac{1}{\alpha _{2}}B(y_{n})y_{n}A(y_{n})\\ y_{n+1}=\alpha _{2}x_{n} \end{array}\right. \end{aligned}$$

with $\mu =\alpha _{1}\cdot \alpha _{2}$. Note that (2.4) is the particular case of (2.1) when $\mu _{1}$ is density-independent and $\mu _{2}=0$. On the other hand, assuming that $\mu _{1}$ and $\mu _{2}$ are density-independent in (2.1), model (2.3) for $k=1$ can also be derived from (2.1) for $p=1$ or for $\mu _{2}=0$.

Model (2.3) implicitly assumes that the lag of the impact on the survival of a previous competition of individuals occurs during the reproduction stage. This could be an oversimplifying assumption for many species. For example, biological observations indicate that the main intraspecific competition episodes occur before reproduction in most tick populations (El-Morshedy and Ruiz-Herrera 2024; Huang et al. 2022; Zhang and Wu 2020). These species require a model formulation with two different delays, namely

2.5 $$\begin{aligned} x_{n+1}=\mu x_{n}+B(x_{n-k_{1}})x_{n-k_{2}}A(x_{n-k_{2}}),\;\;\;n=0,1,2,.... \end{aligned}$$

with $$k_{1},k_{2}\ge 0$$.

## Clark’s equation with delays

This section is divided into two subsections. First, we provide the theoretical results for models (2.3) and (2.5). Then, we biologically interpret the results and compare both models. Specifically, we analyze the influence on the creation of oscillations of two key features of the life cycle of any population - the developmental delay and the time of occurrence of the main episodes of intraspecific competition. In the sequel, we employ the notation $\mathbb {N}$ to denote the set of natural numbers and $\mathbb {N}_{0}=\mathbb {N}\cup \{0\}$.

### Mathematical analysis

We start with a general formulation of Clark’s model with two delays, namely,

3.1 $$\begin{aligned} x_{n+1}=\mu x_{n} +(1-\mu ) F(x_{n-k_{1}},x_{n-k_{2}}),\;\;\;\;\;\;n=0,1,... \end{aligned}$$

with $\mu \in (0,1)$, $k_{1},k_{2}\in \mathbb {N}_{0}$ and $F:[0,+\infty )^{2}\longrightarrow [0,+\infty )$ a continuous map so that

$$\begin{aligned} F((0,+\infty )^{2})\subset (0,+\infty ). \end{aligned}$$

The term $1-\mu$ does not appear in (2.5), but it can be naturally introduced by changing *F* by $\frac{F}{1-\mu }$. We say that $\{x_{n}\}$ is a positive sequence of Eq. (3.1) if it satisfies (3.1) and $x_{n}>0$ for all $n\in \mathbb {N}$. It is worth noting that given $(x_{-\theta },x_{-\theta +1},...,x_{0})\in (0,+\infty )^{\theta +1}$ with $\theta =\max \{k_{1},k_{2}\}$, the sequence obtained from Eq. (3.1) with this initial condition is a positive sequence. We assume the existence of a positive equilibrium ${\bar{x}}>0$ for (3.1), that is, $F({\bar{x}},{\bar{x}})={\bar{x}}$. Moreover, we need the following assumptions:

1. $F(x,y)>y$ for all $(x,y)\in (0,+\infty )^2$ with $x<{\bar{x}}$.
2. $F(x,y)<y$ for all $(x,y)\in (0,+\infty )^2$ with $x>{\bar{x}}$.

A common hypothesis in the study of model (1.1) is

3.2 $$\begin{aligned} h(x)>x\;\;\textrm{if}\;0<x<p\;\;\;\textrm{and}\;\;\;h(x)<x\;\;\textrm{if}\;x>p \end{aligned}$$

where $p>0$ is the nontrivial equilibrium of (1.1). In this case, *p* represents the carrying capacity of (1.1). Generally speaking, conditions **(C1)**–**(C2)** are extensions of (3.2) in higher dimensions. Classical examples of maps satisfying **(C1)**–**(C2)** are $F(x,y)=a y e^{-x}$ and $F(x,y)=\frac{a y}{1+x}$ with $a>1$. In one-dimensional models, condition (3.2) ensures non-extinction and bounded growth of populations. As the next propositions show, **(C1)**–**(C2)** play the same role in (3.1).

#### Proposition 3.1

If **(C1)** and **(C2)** hold, then the positive sequences of (3.1) are bounded.

To prove this result we need an auxiliary result:

#### Lemma 3.1

Let $\{x_{n}\}$ be a positive sequence of (3.1) that admits a subsequence $\{x_{\sigma (n)}\}$ with

$$\begin{aligned} x_{\sigma (n)}\longrightarrow +\infty . \end{aligned}$$

Then, for each $m\in \mathbb {N}$, there exists a subsequence of $\{x_{\sigma (n)}\}$, say $\{x_{\sigma (\theta (n))}\}$, so that

$$\begin{aligned} x_{\sigma (\theta (n))-m}\longrightarrow +\infty . \end{aligned}$$

#### Proof

The sequence $\{x_{\sigma (n)}\}$ tends to $+\infty$ and satisfies the equation

$$\begin{aligned} x_{\sigma (n)}=\mu x_{\sigma (n)-1}+(1-\mu )F(x_{\sigma (n)-k_{1}-1},x_{\sigma (n)-k_{2}-1}). \end{aligned}$$

Therefore, one of the following cases holds:

1. $$x_{\sigma (\tau (n))-1}\longrightarrow +\infty $$ for a suitable $$\tau :\mathbb {N}\longrightarrow \mathbb {N}$$.
2. $$\lim _{n\rightarrow +\infty } F(x_{\sigma (\tau (n))-k_{1}-1},x_{\sigma (\tau (n))-k_{2}-1})= +\infty $$ for a suitable $$\tau :\mathbb {N}\longrightarrow \mathbb {N}$$.

By the continuity of *F* in $[0,+\infty )^2$, this last case can be subdivided into two subcases:

Case 2.1 $x_{\sigma (\tau _{1}(n))-k_{1}-1}\longrightarrow +\infty$ for a suitable $\tau _{1}:\mathbb {N}\longrightarrow \mathbb {N}$.
Case 2.2 $x_{\sigma (\tau _{1}(n))-k_{2}-1}\longrightarrow +\infty$ for a suitable $\tau _{1}:\mathbb {N}\longrightarrow \mathbb {N}$.

Now, we prove that the first case holds provided **case 2.1** holds. From the expression of Eq. (3.1), we deduce that

3.3 $$\begin{aligned} x_{n}\ge \mu ^{k}x_{n-k} \end{aligned}$$

for any positive sequence of (3.1) and $k\in \mathbb {N}$. Recall that $F([0,+\infty )^2)\subset [0,+\infty )$. As a simple application of this, we obtain that the sequences $x_{\sigma (\tau _{1}(n))-k_{1}}$,..., $x_{\sigma (\tau _{1}(n))-k_{1}+(k_{1}-1)}$ tend to $+\infty$. In particular, $x_{\sigma (\tau _{1}(n))-1}\longrightarrow +\infty$. The same happens if we assume that **case 2.2** holds. Since $x_{\sigma (\tau _{1}(n))-1}\longrightarrow +\infty$ for a suitable $\tau _{1}:\mathbb {N}\longrightarrow \mathbb {N}$, we repeat the discussion of cases replacing $x_{\sigma (n)}$ by $x_{\sigma (\tau _{1}(n))-1}$. Arguing as above, we deduce that $x_{\sigma (\tau _{1}(\tau _{2}(n)))-2}\longrightarrow +\infty$ for a suitable $\tau _{2}:\mathbb {N}\longrightarrow \mathbb {N}$. The proof is completed after *m* steps. $\square$

#### Proof of Proposition 3.1

Assume, by contradiction, that there exists a positive sequence of (3.1) so that

$$\begin{aligned} \limsup _{n\longrightarrow +\infty }x_{n}=+\infty . \end{aligned}$$

In such a case, we can take a subsequence $\{x_{\tau (n)+1}\}$ so that

3.4 $$\begin{aligned} x_{\tau (n)+1}=\max \{x_{i}:i\le \tau (n)+1\} \end{aligned}$$

and

3.5 $$\begin{aligned} \lim _{n\longrightarrow +\infty }x_{\tau (n)+1}=+\infty . \end{aligned}$$

From the expression of Eq. (3.1) and using (3.4), we have that

$$\begin{aligned} x_{\tau (n)+1}\le \mu x_{\tau (n)+1}+(1-\mu )F(x_{\tau (n)-k_{1}},x_{\tau (n)-k_{2}}). \end{aligned}$$

Consequently,

3.6 $$\begin{aligned} x_{\tau (n)+1}\le F(x_{\tau (n)-k_{1}},x_{\tau (n)-k_{2}}). \end{aligned}$$

This inequality together with (3.4) lead to

3.7 $$\begin{aligned} x_{\tau (n)-k_{2}} \le F(x_{\tau (n)-k_{1}},x_{\tau (n)-k_{2}}). \end{aligned}$$

Using **(C1)**, **(C2)**, and (3.7), we obtain that

3.8 $$\begin{aligned} x_{\tau (n)-k_{1}}\le {\bar{x}} \end{aligned}$$

for all $n\in \mathbb {N}$. On the other hand, applying Lemma 3.1 with $x_{\tau (n)+1}$ and $m=k_{1}+1$, we deduce the existence of a subsequence, say $x_{\tau (\theta (n))}$, so that

$$\begin{aligned} \lim _{n\longrightarrow +\infty }x_{\tau (\theta (n))-k_{1}}=+\infty , \end{aligned}$$

a contradiction with (3.8). $$\square $$

#### Proposition 3.2

If **(C1)** and **(C2)** hold, then

$$\begin{aligned} \liminf _{n\longrightarrow +\infty } x_{n}>0 \end{aligned}$$

for every positive sequence of (3.1).

#### Proof

Assume, by contradiction, that there is a positive sequence $\{x_{n}\}$ of Eq. (3.1) so that

$$\begin{aligned} \liminf _{n\longrightarrow +\infty } x_{n}=0. \end{aligned}$$

In such a case, we can pick a subsequence $\{x_{\sigma (n)+1}\}$ so that

3.9 $$\begin{aligned} x_{\sigma (n)+1}=\min \{x_{i}:1\le i\le \sigma (n)+1\} \end{aligned}$$

and

3.10 $$\begin{aligned} \lim _{n\longrightarrow +\infty } x_{\sigma (n)+1}=0. \end{aligned}$$

Using (3.9) in Eq. (3.1), we have that

$$\begin{aligned} x_{\sigma (n)+1}\ge \mu x_{\sigma (n)+1}+ (1-\mu ) F(x_{\sigma (n)-k_{1}},x_{\sigma (n)-k_{2}}). \end{aligned}$$

Thus, $x_{\sigma (n)+1}\ge F(x_{\sigma (n)-k_{1}},x_{\sigma (n)-k_{2}})$. By (3.9), we conclude that

3.11 $$\begin{aligned} x_{\sigma (n)-k_{2}}\ge F(x_{\sigma (n)-k_{1}},x_{\sigma (n)-k_{2}}). \end{aligned}$$

Using **(C1)**, **(C2)** and this last condition, we deduce that $x_{\sigma (n)-k_{1}} \ge {\bar{x}}$ for all $n\in \mathbb {N}$. On the other hand, by property (3.3), we obtain that

$$\begin{aligned} x_{\sigma (n)+1}\ge \mu ^{k_{1}+1}x_{\sigma (n)-k_{1}} \end{aligned}$$

and so

$$\begin{aligned} x_{\sigma (n)+1} \ge \mu ^{k_{1}+1} {\bar{x}} \end{aligned}$$

for all $n\in \mathbb {N}$. This is a contradiction with (3.10).$\square$

The following result provides a useful estimation of the positive sequences of (3.1).

#### Lemma 3.2

Let $\{x_{n}\}$ be a positive sequence of (3.1). Then, for each $m\in \mathbb {N}$,

3.12 $$\begin{aligned} \left\{ \begin{array}{lll} x_{n+1}\le \mu ^{m+1} x_{n-m}+(1-\mu ^{m+1}) F(\xi _{nk_{1}},\xi _{nk_{2}})\\ x_{n+1}\ge \mu ^{m+1} x_{n-m}+(1-\mu ^{m+1}) F(\kappa _{nk_{1}},\kappa _{nk_{2}}) \end{array}\right. \end{aligned}$$

where $$\xi _{nk_{1}}, \xi _{nk_{2}},\kappa _{nk_{1}},\kappa _{nk_{2}}\in [A_{n},B_{n}]$$ with

$$\begin{aligned} A_{n}=\min \{x_{n-\theta -m},...,x_{n}\}, \end{aligned}$$

$$\begin{aligned} B_{n}=\max \{x_{n-\theta -m},...,x_{n}\}, \end{aligned}$$

$$\theta =\max \{k_{1},k_{2}\}$$.

#### Proof

We prove these inequalities for the case $m=1$. By the expression of (3.1), we deduce that

$$\begin{aligned} x_{n+1}=\mu x_{n}+(1-\mu ) F(x_{n-k_{1}},x_{n-k_{2}}). \end{aligned}$$

Inserting

$$\begin{aligned} x_{n}=\mu x_{n-1}+(1-\mu ) F(x_{n-k_{1}-1},x_{n-k_{2}-1}) \end{aligned}$$

in the previous equality, we obtain that

$$\begin{aligned} x_{n+1}=\mu ^2 x_{n-1}+(1-\mu ) F(x_{n-k_{1}},x_{n-k_{2}})+\mu (1-\mu )F(x_{n-k_{1}-1},x_{n-k_{2}-1}). \end{aligned}$$

Let

$$\begin{aligned} \beta _{1}=\min \{F(x_{n-k_{1}},x_{n-k_{2}}),F(x_{n-k_{1}-1},x_{n-k_{2}-1})\} \end{aligned}$$

and

$$\begin{aligned} \beta _{2}=\max \{F(x_{n-k_{1}},x_{n-k_{2}}),F(x_{n-k_{1}-1},x_{n-k_{2}-1})\}. \end{aligned}$$

Obviously,

$$\begin{aligned} x_{n+1}\le \mu ^{2} x_{n-1}+(1-\mu ^{2})\beta _{2} \end{aligned}$$

and

$$\begin{aligned} x_{n+1}\ge \mu ^{2} x_{n-1}+(1-\mu ^{2})\beta _{1}. \end{aligned}$$

At this moment, the conclusion is clear for $m=1$. The proof follows after a simple induction. $\square$

Our criteria for global attraction consist of constructing a suitable scalar equation so that the global attraction in the latter implies the global attraction for (3.1). The next theorem is a preliminary and technical step towards this construction.

#### Theorem 3.1

Assume that **(C1)** and **(C2)** hold and ${\bar{x}}$ is the unique positive equilibrium of (3.1). Suppose that there is a positive sequence $\{x_{n}\}$ of (3.1) that does not converge to ${\bar{x}}$. Then, there are six positive constants $L,S,L_{1},L_{2},S_{1},S_{2}$ with the following properties:

1. $$L=\text {lim inf}_{n\rightarrow +\infty } x_{n}$$ and $$S=\text {lim sup}_{n\rightarrow +\infty } x_{n}$$.
2. $$L,S\in (\mu ^{k_{1}+1}{\bar{x}},+\infty )$$ with $$L<S$$.
3. $L_{1},L_{2},S_{1},S_{2}$ belong to the interval [*L*, *S*].
4. $$L\ge \mu ^{k_{1}+1} {\bar{x}}+(1-\mu ^{k_{1}+1}) F(L_{1},L_{2}).$$
5. $$S\le \mu ^{k_{1}+1} {\bar{x}}+(1-\mu ^{k_{1}+1}) F(S_{1},S_{2}).$$

#### Proof

Let $L=\liminf _{n\rightarrow +\infty } x_{n}$ and $S=\limsup _{n\rightarrow +\infty } x_{n}$. By Propositions 3.1 and 3.2, we deduce that $L>0$ and $S\in (0,+\infty )$. Since (3.1) has a unique positive equilibrium, we obtain $L<S$. On the other hand, we can take two subsequences of $\{x_{n}\}$, say $\{x_{\sigma (n)}\}$ and $\{x_{\tau (n)}\}$, so that $L=\lim _{n\rightarrow +\infty } x_{\sigma (n)}$ and $S=\lim _{n\rightarrow +\infty } x_{\tau (n)}$. Using that $\{x_{n}\}$ is bounded, we deduce the existence of six positive constants ${\widetilde{L}}_{1},{\widetilde{L}}_{2},{\widetilde{S}}_{1},{\widetilde{S}}_{2}, {\widetilde{L}}_{3},{\widetilde{S}}_{3}$ with the following properties:

1. ${\widetilde{L}}_{1},{\widetilde{L}}_{2},{\widetilde{S}}_{1},{\widetilde{S}}_{2},{\widetilde{L}}_{3},{\widetilde{S}}_{3}$ belong to the interval [*L*, *S*].
2. $$x_{\sigma (n)}\longrightarrow L$$, $$x_{\sigma (n)-1}\longrightarrow {\widetilde{L}}_{3}$$, $$x_{\sigma (n)-k_{1}-1}\longrightarrow {\widetilde{L}}_{1}$$, $$x_{\sigma (n)-k_{2}-1}\longrightarrow {\widetilde{L}}_{2}$$, $$x_{\tau (n)}\longrightarrow S$$, $$x_{\tau (n)-1}\longrightarrow {\widetilde{S}}_{3}$$, $$x_{\tau (n)-k_{1}-1}\longrightarrow {\widetilde{S}}_{1}$$ and $$x_{\tau (n)-k_{2}-1}\longrightarrow {\widetilde{S}}_{2}$$.

Evaluating Eq. (3.1) at $x_{\sigma (n)}$ and $x_{\tau (n)}$, we obtain that

$$\begin{aligned} x_{\sigma (n)}=\mu x_{\sigma (n)-1}+(1-\mu ) F(x_{\sigma (n)-k_{1}-1},x_{\sigma (n)-k_{2}-1}), \end{aligned}$$

$$\begin{aligned} x_{\tau (n)}=\mu x_{\tau (n)-1}+(1-\mu ) F(x_{\tau (n)-k_{1}-1},x_{\tau (n)-k_{2}-1}), \end{aligned}$$

respectively. Making $n\rightarrow +\infty$, the equations

3.13 $$\begin{aligned} L=\mu {\widetilde{L}}_{3}+ (1-\mu ) F({\widetilde{L}}_{1},{\widetilde{L}}_{2}) \end{aligned}$$

and

3.14 $$\begin{aligned} S=\mu {\widetilde{S}}_{3}+ (1-\mu ) F({\widetilde{S}}_{1},{\widetilde{S}}_{2}) \end{aligned}$$

are satisfied. Using **(D1)**, we have that

3.15 $$\begin{aligned} \left\{ \begin{array}{lll} S\le F({\widetilde{S}}_{1},{\widetilde{S}}_{2})\\ L\ge F({\widetilde{L}}_{1},{\widetilde{L}}_{2}). \end{array}\right. \end{aligned}$$

We divide the rest of the proof into three steps:*Step 1:* We prove that $L\le {\bar{x}}$ and $S\ge {\bar{x}}$.Assume, by contradiction, that $L>{\bar{x}}$. In such a case, ${\widetilde{S}}_{1},{\widetilde{S}}_{2}>{\bar{x}}$ by **(D1)**. On the other hand, using the first inequality in (3.15), we deduce that ${\widetilde{S}}_{2}\le F({\widetilde{S}}_{1},{\widetilde{S}}_{2})$. Now, conditions **(C1)** and **(C2)** imply that ${\widetilde{S}}_{1}\le {\bar{x}}$. This is a contradiction because ${\widetilde{S}}_{1}\ge L>{\bar{x}}$. We can prove that $S\ge {\bar{x}}$ in a similar manner.

*Step 2:* We prove that ${\widetilde{L}}_{1}\ge {\bar{x}}$ and ${\widetilde{S}}_{1}\le {\bar{x}}$.By the second inequality in (3.15) and **(D1)**, we know that $F({\widetilde{L}}_{1},{\widetilde{L}}_{2})-{\widetilde{L}}_{2}\le 0$. Therefore, **(C2)** leads to $\widetilde{L_1}\ge {\bar{x}}$. We can deduce that ${\widetilde{S}}_{1}\le {\bar{x}}$ in a similar manner.

*Step 3:* Conclusion.We have that

$$\begin{aligned} x_{\sigma (n)}=\mu x_{\sigma (n)-1}+(1-\mu ) F(x_{\sigma (n)-k_{1}-1},x_{\sigma (n)-k_{2}-1}). \end{aligned}$$

Using Lemma 3.2 and the second inequality in (3.12) with $m=k_{1}$, we conclude the existence of two bounded sequences $\{\kappa _{nk_{1}}\}, \{\kappa _{nk_{2}}\}$ so that

3.16 $$\begin{aligned} x_{\sigma (n)}\ge \mu ^{k_{1}+1} x_{\sigma (n)-1-k_{1}}+(1-\mu ^{k_{1}+1}) F(\kappa _{nk_{1}}, \kappa _{nk_{2}}). \end{aligned}$$

It is not restrictive to assume that $\{\kappa _{nk_{1}}\}, \{\kappa _{nk_{2}}\}$ tend to $L_{1},L_{2}$ with $L_{1},L_{2}\in [L,S]$, respectively. Recall that $x_{\sigma (n)-1-k_{1}}\longrightarrow {\widetilde{L}}_{1}$. Making $n\longrightarrow +\infty$ in (3.16) and using the estimates in the Step 2, we obtain that

3.17 $$\begin{aligned} L\ge \mu ^{k_{1}+1} {\bar{x}}+(1-\mu ^{k_{1}+1}) F(L_{1},L_{2}). \end{aligned}$$

Arguing similarly, we deduce the existence of two constants $S_{1},S_{2}\in [L,S]$ so that

3.18 $$\begin{aligned} S\le \mu ^{k_{1}+1} {\bar{x}}+(1-\mu ^{k_{1}+1}) F(S_{1},S_{2}). \end{aligned}$$

We stress that the inequality in (3.17) implies that $L>\mu ^{k_{1}+1} {\bar{x}}$. $\square$

The method of proof and the conclusions derived in Theorem 3.1 are reminiscent of the classical “decomposing+embedding” method, (see Enciso et al. 2006; Kulenović and Merino 2006; Smith 2006, 2008). Roughly speaking, the idea of this method is to decompose the equation into its increasing and decreasing parts and embed the system into a larger monotone system. The trick is that one recovers the original system by restricting the larger system on the diagonal. In the case of model (3.1), we should consider larger systems of the form

$$\begin{aligned} \left\{ \begin{array}{lll} x_{n+1}=\mu x_{n}+ (1-\mu ) F(y_{n-k_{1}},x_{n-k_{2}})\\ y_{n+1}=\mu y_{n}+ (1-\mu ) F(y_{n-k_{1}},x_{n-k_{2}}), \end{array}\right. \end{aligned}$$

studying its monotonicity properties and the non-existence of fixed points different from $({\bar{x}},{\bar{x}})$. In this last step, system (3.13) and (3.14) would appear.

We take a constant birth rate *a* in the applications (see Yu and Li 2022). We analyze, therefore, model

3.19 $$\begin{aligned} x_{n+1}=\mu x_{n}+(1-\mu ) b(x_{n-k_{1}}) a x_{n-k_{2}}. \end{aligned}$$

In this case, $F(x,y)=a y b(x)$. The next result describes precisely the construction of the scalar equation that guarantees the global attraction in (3.1). Before its precise statement, we recall a result on scalar equations for the reader’s convenience.

#### Lemma 3.3

(Lemma 2.5 in El-Morshedy and López (2008)) Let $\varphi :(\omega _{1},\omega _{2})\longrightarrow (\omega _{1},\omega _{2})$ be a continuous function. Assume that ${\bar{x}}\in (\omega _{1},\omega _{2})$ with $\varphi ({\bar{x}})={\bar{x}}$ is globally attracting for equation

$$\begin{aligned} x_{n+1}=\varphi (x_{n}) \end{aligned}$$

in $(\omega _{1},\omega _{2})$, that is, for all $x_{0}\in (\omega _{1},\omega _{2})$, $\lim _{n \rightarrow +\infty }\varphi ^{n}(x_{0})={\bar{x}}$ with $\varphi ^{n}=\varphi \, \circ {\mathop {\cdots }\limits ^{n)}} \circ \, \varphi$. Then, there are no intervals $[L,S]\subset (\omega _{1},\omega _{2})$ with $L<S$ so that $[L,S]\subset \varphi ([L,S])$.

#### Theorem 3.2

Assume that $b:[0,+\infty )\longrightarrow (0,+\infty )$ is a strictly decreasing function that satisfies the following conditions:

1. There is ${\bar{x}}>0$ so that $b({\bar{x}})=\frac{1}{a}$.
2. $$b(\mu ^{k_{1}+1}{\bar{x}})<\frac{1}{a(1-\mu ^{k_{1}+1})}$$.

If ${\bar{x}}>0$ is an attractor in $(\mu ^{k_{1}+1} {\bar{x}},+\infty )$ for the equation

3.20 $$\begin{aligned} z_{n+1}=\frac{\mu ^{k_{1}+1} {\bar{x}}}{1-a(1-\mu ^{k_{1}+1})b(z_{n})},\;\;\;\;\;\;n=0,1,... \end{aligned}$$

then

$$\begin{aligned} \lim _{n\rightarrow +\infty } x_{n}={\bar{x}} \end{aligned}$$

for every positive sequence $\{x_{n}\}$ of model (3.19).

#### Proof

Using that *b* is strictly decreasing and **(B1)**, it is clear that *F* satisfies **(C1)** and **(C2)**. Now, we assume, by contradiction, that there is a positive sequence $\{x_{n}\}$ of (3.19) so that . By Theorem 3.1, there are six positive constants $L,S, L_{1},L_{2},S_{1},S_{2}$ with the following properties:

- $$L=\liminf _{n\rightarrow +\infty } x_{n}$$ and $$S=\limsup _{n\rightarrow +\infty } x_{n}$$.
- $$L,S\in (\mu ^{k_{1}+1} {\bar{x}},+\infty )$$ with $$L<S$$.
- $L_{1},L_{2},S_{1},S_{2}$ belong to the interval [*L*, *S*].
- The inequalities
  $$\begin{aligned} \left\{ \begin{array}{lll} S\le \mu ^{k_{1}+1} {\bar{x}}+(1-\mu ^{k_{1}+1}) S_{2} a \;b(S_{1})\\ L\ge \mu ^{k_{1}+1} {\bar{x}}+(1-\mu ^{k_{1}+1}) L_{2} a\; b(L_{1}) \end{array}\right. \end{aligned}$$
  are satisfied.

Using that $S_{2},L_{2}\in [L,S]$, we obtain

$$\begin{aligned} \left\{ \begin{array}{lll} S\le \mu ^{k_{1}+1} {\bar{x}}+(1-\mu ^{k_{1}+1}) S a\; b(S_{1})\\ L\ge \mu ^{k_{1}+1} {\bar{x}}+(1-\mu ^{k_{1}+1}) L a \;b(L_{1}). \end{array}\right. \end{aligned}$$

Therefore, after simple manipulations together with **(B2)**, we arrive at

$$\begin{aligned} \left\{ \begin{array}{lll} S\le \frac{\mu ^{k_{1}+1}{\bar{x}}}{ 1-(1-\mu ^{k_{1}+1})a\;b(S_{1})}\\ L\ge \frac{\mu ^{k_{1}+1}{\bar{x}}}{ 1-(1-\mu ^{k_{1}+1}) a\; b(L_{1})}. \end{array}\right. \end{aligned}$$

The function

$$\begin{aligned} \varphi (x)=\frac{\mu ^{k_{1}+1} {\bar{x}}}{ 1-(1-\mu ^{k_{1}+1})a\;b(x)} \end{aligned}$$

is well defined for all $x>\mu ^{k_{1}+1} {\bar{x}}$ by **(B2)**. Notice that the last system of inequalities implies that $[L,S]\subset \varphi ([L,S])$. On the other hand, Lemma 3.3 claims that if ${\bar{x}}$ is a global attractor of

$$\begin{aligned} z_{n+1}=\varphi (z_{n}) \end{aligned}$$

in $(\mu ^{k_{1}+1}{\bar{x}},+\infty )$, then there are no intervals $[L,S]\subset (\mu ^{k_{1}+1}{\bar{x}},+\infty )$ with $L<S$ so that $[L,S]\subset \varphi ([L,S])$. We have obtained a contradiction. $\square$

If the delay of the impact on the survival of a previous competition of individuals occurs during the reproduction period, model (3.1) has a unique delay. In such a case, we have

3.21 $$\begin{aligned} x_{n+1}=\mu x_{n}+(1-\mu ) G(x_{n-k}) \end{aligned}$$

with $\mu \in (0,1)$, $k\in \mathbb {N}_{0}$, and $G:[0,+\infty )\longrightarrow [0,+\infty )$ a continuous map so that

$$\begin{aligned} G((0,+\infty ))\subset (0,+\infty ). \end{aligned}$$

Conditions **(C1)** and **(C2)** now read in the following manner: There exists ${\bar{x}}>0$ so that

1. $$G(x)>x$$ if $$x\in (0,{\bar{x}})$$,
2. $$G(x)<x$$ if $$x\in ({\bar{x}},+\infty )$$.

Note that **(C1’)** and **(C2’)** imply that ${\bar{x}}$ is the unique positive equilibrium of (3.21). Actually, **(C1’)**-**(C2’)** are common assumptions in one-dimensional models with ${\bar{x}}$ the carrying capacity. Repeating and adapting the arguments of the previous subsection, we can deduce the following result:

#### Theorem 3.3

Assume that **(C1’)** and **(C2’)** hold. Suppose that there is a positive sequence $\{x_{n}\}$ of (3.21) that does not converge to ${\bar{x}}$. Then, there are four constants $L,S,L_{1},S_{1}$ with the following properties:

1. $$L=\liminf _{n\rightarrow +\infty } x_{n}$$ and $$S=\limsup _{n\rightarrow +\infty } x_{n}$$.
2. $$L,S\in (\mu ^{k+1}{\bar{x}},+\infty )$$ with $$L<S$$.
3. $L_{1},S_{1}$ belong to the interval [*L*, *S*].
4. $$L\ge \mu ^{k+1} {\bar{x}}+(1-\mu ^{k+1}) G(L_{1}).$$
5. $$S\le \mu ^{k+1} {\bar{x}}+(1-\mu ^{k+1}) G(S_{1}).$$

From this theorem, we derive the following practical criterion of attraction in (3.21) when $G(x)=b(x)a x$. As above, we suppose that $a>0$ and $b:[0,+\infty )\longrightarrow [0,+\infty )$ is a continuous function with $b((0,+\infty ))\subset (0,+\infty )$.

#### Theorem 3.4

Assume that $b:[0,+\infty )\longrightarrow (0,+\infty )$ is a strictly decreasing function with the following condition:

1. There is ${\bar{x}}>0$ so that $b({\bar{x}})=\frac{1}{a}$.

If ${\bar{x}}>0$ is a global attractor in $(\mu ^{k+1} {\bar{x}},+\infty )$ for the equation

3.22 $$\begin{aligned} z_{n+1}=\mu ^{k+1}{\bar{x}}+(1-\mu ^{k+1}) G(z_{n})\;\;\;\;\;\;n=0,1,..., \end{aligned}$$

then

$$\begin{aligned} \lim _{n\rightarrow +\infty } x_{n}={\bar{x}} \end{aligned}$$

for every positive sequence $\{x_{n}\}$ of model (3.21).

#### Proof

It is clear that *G* satisfies **(C1’)** and **(C2’)** using that *b* is strictly decreasing together with **(B1)**. Now, we assume, by contradiction, there is a positive sequence $\{x_{n}\}$ so that . By Theorem 3.3, there are four constants $L,S, L_{1},S_{1}$ with the following properties:

- $$L=\liminf _{n\rightarrow +\infty } x_{n}$$ and $$S=\limsup _{n\rightarrow +\infty } x_{n}$$.
- $$L,S\in (\mu ^{k+1} {\bar{x}},+\infty )$$ with $$L<S$$.
- $L_{1},S_{1}$ belong to the interval [*L*, *S*].
- The inequalities
  $$\begin{aligned} \left\{ \begin{array}{lll} S\le \mu ^{k+1} {\bar{x}}+(1-\mu ^{k+1}) G(S_{1})\\ L\ge \mu ^{k+1} {\bar{x}}+(1-\mu ^{k+1})G(L_{1}). \end{array}\right. \end{aligned}$$
  are satisfied.

The function

$$\begin{aligned} {\widetilde{\varphi }}(x)=\mu ^{k+1} {\bar{x}}+(1-\mu ^{k+1}) a x b(x) \end{aligned}$$

satisfies that $[L,S]\subset {\widetilde{\varphi }}([L,S])$. On the other hand, Lemma 3.3 claims that if ${\bar{x}}$ is a global attractor of

$$\begin{aligned} z_{n+1}={\widetilde{\varphi }}(z_{n}) \end{aligned}$$

in $z_{0}\in (\mu ^{k+1}{\bar{x}},+\infty )$, then there are no intervals $[L,S]\subset (\mu ^{k+1}{\bar{x}},+\infty )$ with $L<S$ so that $[L,S]\subset {\widetilde{\varphi }}([L,S])$. This contradiction completes the proof. $\square$

### One delay or two delays?

This subsection aims to translate the abstract framework developed in Sect. 3.1 into an applied one. For simplicity, we illustrate our results using two classical functions in theoretical ecology: The Beverton–Holt function and the Ricker function. As stressed by Brannstrom and Sumpter (2005), populations with random spatial distributions and scramble competition exhibit simple Ricker dynamics. This type of competition for resources is experienced by many species, including most microbes, fishes, invertebrates, and amphibians. By contrast, the Beverton–Holt dynamics are related to contest competition (see Brannstrom and Sumpter 2005). Since Theorems 3.1 and 3.3 are written in terms of the attraction of a scalar equation (without delay), we recall a basic result in discrete dynamics for the reader’s convenience, (see El-Morshedy and López 2008; Singer 1978 for more details).

#### Proposition 3.3

Assume that $\Phi :(\omega ,+\infty )\rightarrow (\omega ,+\infty )$ is a continuous and strictly decreasing or unimodal function of class $\mathcal {C}^{3}$ in $(\omega ,+\infty )$ with $\omega \in \mathbb {R}$. Suppose that $\Phi$ has a fixed point ${\overline{x}}$ with $\Phi '({\overline{x}})\ge -1$. If $S(\Phi )(x)\le 0$ on $(\omega ,+\infty )$ where

$$\begin{aligned} S(\Phi )(x)=\frac{\Phi '''(x)}{\Phi '(x)}-\frac{3}{2}\left( \frac{\Phi ''(x)}{\Phi '(x)}\right) ^2, \end{aligned}$$

for all $x\in (\omega ,+\infty )$ with $\Phi '(x)\not =0$, then, ${\overline{x}}$ is a global attractor in $(\omega ,+\infty )$ for the difference equation

3.23 $$\begin{aligned} x_{n+1}=\Phi (x_{n}). \end{aligned}$$

**Clark’s model with the Beverton–Holt function**Consider

3.24 $$\begin{aligned} x_{n+1}=\mu x_{n}+\frac{a x_{n-k_{2}}}{1+x_{n-k_{1}}} \end{aligned}$$

with $\mu \in (0,1)$, $a>1-\mu$, and $k_{1},k_{2}\in \mathbb {N}_{0}$. In terms of Theorems 3.2 and 3.4, $b(x)=\frac{1}{(1-\mu )(1+x)}$ and ${\bar{x}}=\frac{a}{1-\mu }-1$ for model (3.24).

#### Theorem 3.5

(i) If $k_{1}=k_{2}$, then $\lim _{n\rightarrow +\infty } x_{n}={\bar{x}}$ for every positive sequence $\{x_{n}\}$ of model (3.24).
(ii) If $k_{1}\not =k_{2}$ and
  3.25 $$\begin{aligned} \frac{1}{\mu ^{k_{1}+1}}<\left( \frac{a}{a+\mu -1}+1\right) , \end{aligned}$$
  then $\lim _{n\rightarrow +\infty } x_{n}={\bar{x}}$ for every positive sequence $\{x_{n}\}$ of model (3.24).

#### Proof

**(i)** Let $k=k_{1}=k_{2}$. It is clear that ${\widetilde{\varphi }}(z)=\mu ^{k+1}{\bar{x}}+(1-\mu ^{k+1})\frac{a z}{(1-\mu )(1+z)}$ is strictly increasing and bounded in $(0,+\infty )$. Moreover, ${\bar{x}}$ is the unique fixed point of ${\widetilde{\varphi }}$ in $(0,+\infty )$. Thus, it is straightforward to prove that ${\bar{x}}$ is an attractor in $(0,+\infty )$ for the difference equation

$$\begin{aligned} z_{n+1}={\widetilde{\varphi }}(z_{n}). \end{aligned}$$

The conclusion now follows from Theorem 3.4.

**(ii)** The condition $b(\mu ^{k_{1}+1}{\bar{x}})<\frac{1}{a(1-\mu ^{k_{1}+1})}$ is $\frac{1}{1+\mu ^{k_{1}+1}(\frac{a}{1-\mu }-1)}<\frac{1-\mu }{a(1-\mu ^{k_{1}+1})}.$ This last expression is equivalent to

3.26 $$\begin{aligned} \frac{a}{1-\mu }(1-\mu ^{k_{1}+1})<1+\mu ^{k_{1}+1}\left( \frac{a}{1-\mu }-1\right) \end{aligned}$$

and (3.25). Now, define

$$\begin{aligned} \varphi (x)=\frac{\mu ^{k_{1}+1}(\frac{a}{1-\mu }-1)}{1-a(1-\mu ^{k_{1}+1})b(x)}. \end{aligned}$$

After a simple computation, we have that $\varphi '({\bar{x}})=\frac{-(\frac{a}{1-\mu }-1) (1-\mu ^{k_{1}+1})}{\frac{a}{1-\mu } \mu ^{k_{1}+1}}$. Thus, $\varphi '({\bar{x}})> -1$ can be written as $(\frac{a}{1-\mu }-1)< \mu ^{k_{1}+1}(2 \frac{a}{1-\mu }-1)$. This last condition coincides with (3.26) and (3.25). Moreover, we have that $\varphi$ is strictly decreasing and $S(\varphi )(x)=0$ for all $x\in (\mu ^{k_{1}+1}{\bar{x}},+\infty )$, (see Singer 1978). The conclusion now follows from Proposition 3.3 and Theorem 3.2. $\square$

**Clark’s model with the Ricker function**

Consider

3.27 $$\begin{aligned} x_{n+1}=\mu x_{n}+a x_{n-k_{2}} e^{-x_{n-k_{1}}} \end{aligned}$$

with $\mu \in (0,1)$, $a>1-\mu$, and $k_{1},k_{2}\in \mathbb {N}_{0}$. In terms of Theorems 3.2 and 3.4, $b(x)=\frac{e^{-x}}{1-\mu }$ and ${\overline{x}}=\ln \frac{a}{1-\mu }$ for model (3.27).

#### Theorem 3.6

(i) If $$k_{1}=k_{2}$$ and
  3.28 $$\begin{aligned} a\le (1-\mu )e^{ 1+\frac{1}{1-\mu ^{k_{1}+1}}}, \end{aligned}$$
  then $\lim _{n\rightarrow +\infty } x_{n}={\bar{x}}$ for every positive sequence $\{x_{n}\}$ of model (3.27).
(ii) If $k_{1}\not =k_{2}$ and
  3.29 $$\begin{aligned} a< (1-\mu )e^{\frac{\mu ^{k_{1}+1}}{1-\mu ^{k_{1}+1}}}, \end{aligned}$$
  then $\lim _{n\rightarrow +\infty }x_{n}={\bar{x}}$ for every positive sequence $\{x_{n}\}$ of model (3.27).

#### Proof

**(i)** Let $k=k_{1}=k_{2}$. Consider ${\widetilde{\varphi }}(x)=\mu ^{k+1}{\bar{x}}+(1-\mu ^{k+1})\frac{a}{1-\mu } x e^{-x}$. We list some basic properties of ${\widetilde{\varphi }}$.

- ${\widetilde{\varphi }}$ is a unimodal map with the critical point at 1.
- ${\widetilde{\varphi }}$ has a unique fixed point ${\bar{x}}=\ln \frac{a}{1-\mu }$.
- $$S({\widetilde{\varphi }})(x)<0$$ for all $$x\in (0,+\infty )\backslash \{1\}$$.
- $${\widetilde{\varphi }}'({\bar{x}})=(1-\mu ^{k+1})(1-\ln \frac{a}{1-\mu })$$.

Next, we discuss the attraction in $(0,+\infty )$ of ${\bar{x}}$ for the difference equation

3.30 $$\begin{aligned} z_{n+1}={\widetilde{\varphi }}(z_{n}). \end{aligned}$$

If ${\bar{x}}\in (0,1)$, ${\bar{x}}$ is an attractor in $(0,+\infty )$ for (3.30) because ${\widetilde{\varphi }}$ is strictly increasing in (0, 1) and ${\widetilde{\varphi }}((0,+\infty ))\subset (0,1)$. If ${\bar{x}}\ge 1$, we can deduce the same by using condition $\ln \frac{a}{1-\mu }\le 1+\frac{1}{1-\mu ^{k+1}}$ and Proposition 3.3. The proof of **(i)** now follows from Theorem 3.4.

**(ii)** After taking logarithms, we notice that $b(\mu ^{k_{1}+1}\ln \frac{a}{1-\mu })< \frac{1}{a(1-\mu ^{k_{1}+1})}$ is equivalent to

3.31 $$\begin{aligned} \ln \frac{a}{1-\mu }< \frac{-\ln (1-\mu ^{k_{1}+1})}{1-\mu ^{k_{1}+1}}. \end{aligned}$$

On the other hand, $\varphi (x)=\frac{\mu ^{k_{1}+1}\ln \frac{a}{1-\mu }}{1-\frac{a}{1-\mu }(1-\mu ^{k_{1}+1})e^{-x}}$ satisfies that

3.32 $$\begin{aligned} \varphi '\left( \ln \frac{a}{1-\mu }\right) =\frac{-(1-\mu ^{k_{1}+1})\ln \frac{a}{1-\mu }}{\mu ^{k_{1}+1}}. \end{aligned}$$

Note that $\ln \frac{a}{1-\mu }<\frac{\mu ^{k_{1}+1}}{1-\mu ^{k_{1}+1}}$ (or (3.29)) implies that $\varphi '(\ln \frac{a}{1-\mu })> -1$ and (3.31). The proof of **(ii)** now follows from Proposition 3.3 and Theorem 3.2 since $\varphi$ is strictly decreasing with $S(\varphi )(x)<0$ for all $x\in (\mu ^{k_{1}+1}\ln \frac{a}{1-\mu },+\infty )$. To see that $S(\varphi )(x)<0$, we first note that $\varphi =\varphi _{1}\circ b$ with $\varphi _{1}(x)=\frac{\mu ^{k_{1}+1}\ln \frac{a}{1-\mu }}{1-\frac{a}{1-\mu }(1-\mu ^{k_{1}+1})x}$. Moreover, following the composition rule (Singer (1978)), we have that $S(\varphi )(x)=S(\varphi _{1}(B(x)))\cdot (b'(x))^{2}+S(b(x))$. Finally, we observe that $S(\varphi _{1})(x)=0$ and $S(b(x))<0$ for all $x>0$. $\square$

**Fig. 1:**
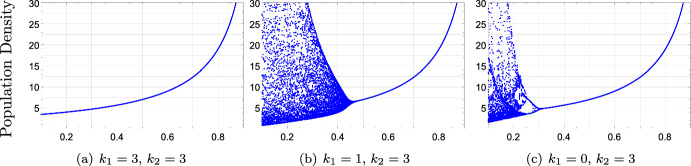
Bifurcation diagrams of model (3.24) with $\mu$ as a bifurcation parameter. Fixed parameter $a=4$. The presence of two delays can create oscillations

Theorems 3.5 suggests two biological lessons. First, the presence of two delays typically produces oscillations in model (3.24). In other words, populations under contest competition are prone to exhibit oscillations when the main intraspecific competition episodes do not occur during the reproduction season. For $a=4$, model (3.24) has simple dynamics when $k_{1}=k_{2}$, (Fig. 1a). However, there are oscillations when $k_{1}\not =k_{2}$, (Fig. 1 b,c). Second, if the adult’s probability of surviving is high ($\mu$ close to 1) and the birth rate is not too high (upper bound is provided in (3.25)), the population displays simple dynamical behaviors, (Fig. 1 b, c). This lesson is also valid for model (3.27), (see Theorem 3.6). Under this ecological context, simple dynamics are also promoted when the main intraspecific competition episodes occur close to the adult stage, (small values of $k_{1}$). Note that in Fig. 1, the range of values of $\mu$ in which the model has simple dynamics is greater when $k_{1}=0$ and $k_{2}=3$ than when $k_{1}=1$ and $k_{2}=3$.

**Fig. 2:**
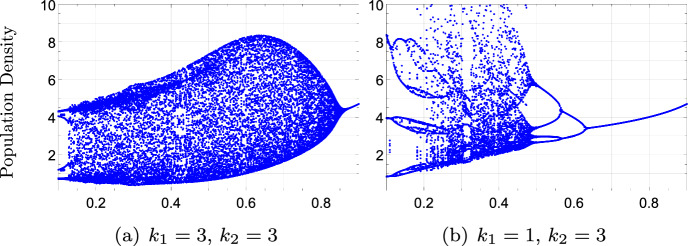
Bifurcation diagrams of model (3.27) with $\mu$ as a bifurcation parameter. Fixed parameter $a=e^{2.4}$. In contrast to model (3.24), the presence of two delays can sometimes simplify the dynamical behavior in (3.27)

Apart from these biological results, it is challenging to describe the dynamical behavior of (3.24) and (3.27) for any value of $a,\mu$, $k_{1}$ and $k_{2}$. Actually, the presence of two delays is a source of new dynamical behaviors, especially under scramble competition. For example, the presence of two delays can stabilize the dynamical behavior of (3.27). In Fig. 2, for $a=e^{2.4}$ and $k_{1}=k_{2}=3$, there are oscillations when $\mu \in (0,0.85)$ but for $k_{1}=1$ and $k_{2}=3$, there are no oscillations for $\mu >0.65$.

**Fig. 3:**
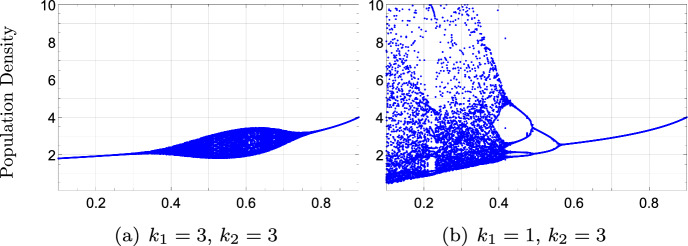
Bifurcation diagrams of model (3.27) with $\mu$ as a bifurcation parameter. Fixed parameter $a=e^{1.4}$. The presence of two delays does not maintain the dynamical behavior of the models

On the other hand, Eq. (3.27) with $k_{1}=k_{2}$ can exhibit bubbling patterns, that is, the equilibrium loses its stability through a Hopf bifurcation and the equilibrium regains its stability for large values of $\mu$, (Fig. 3a). However, model (3.27) for the same values of *a* and $k_{1}=1$, $k_{2}=3$ exhibits a common cascade of bifurcation.

## Stage-structured models

Yu and Li (2022) employed a particular formulation of model (2.1) to describe the dynamical behavior of mosquito populations. Specifically, they analyzed the model

4.1 $$\begin{aligned} \left\{ \begin{array}{lll} J_{n+1}=\frac{a k A_{n}}{1+\eta J_{n}}+ \frac{\gamma k J_{n}}{1+\eta J_{n}}\\[2ex] A_{n+1}=\frac{\beta J_{n}}{1+\eta J_{n}}+\mu A_{n} \end{array}\right. \end{aligned}$$

where $a,\eta >0$; $k,\mu \in (0,1)$; $\gamma ,\beta \in [0,1]$. Mosquitoes have four life stages during their complete development: egg, larva, pupa, and adult. The common crowdings occur in water during the first three stages. Moreover, adults live in the air, and their interspecific competition is generally weak and negligible. Motivated by these two facts, we have grouped the individuals of the first three stages in water as one group and denoted them as a juvenile group ($J_{n}$). Thus, the wild mosquito population is divided into only two groups in (4.1), juveniles and adults. In this model, $\mu$ is the survival probability of adult mosquitoes. The terms $\frac{a k}{1+\eta J_{n}}$ and $\frac{\gamma k}{1+\eta J_{n}}$ represent the natality and survival rates during the juvenile stage, respectively. Analogously, $\frac{\beta J_{n}}{1+\eta J_{n}}$ denotes the density of juvenile individuals that pass to the adult stage.

Yu and Li (2022) proved that when (4.1) admits an equilibrium $E_{*}=(J_{*},A_{*})$ with $J_{*}>0$ and $A_{*}>0$, then $E_{*}$ is locally asymptotically stable. The main result of this section claims that $E_{*}$ is always globally asymptotically stable. This result has deep ecological repercussions. Although (4.1) was proposed to study the dynamical behavior of wild mosquitoes, this model could be valid for a broad spectrum of species. In fact, we only assume that the population is divided into two stage groups and contest competition in both groups. The main conclusion is, therefore, that contest competition alone can buffer any oscillation, independently of the reproduction strategy and developmental rate.

In the next subsection, we analyze

4.2 $$\begin{aligned} \left\{ \begin{array}{lll} J_{n+1}=\frac{a k A_{n}}{1+\eta J_{n}}+ \frac{\gamma k J_{n}}{1+\eta J_{n}}\\[2ex] A_{n+1}=f(J_{n})+\mu A_{n} \end{array}\right. \end{aligned}$$

where $a,\eta >0$; $k,\mu \in (0,1)$; $\gamma \in [0,1]$. This model is slightly more general than (4.1). The function $f:[0,+\infty )\longrightarrow [0,+\infty )$ represents the adult’s recruitment. We assume that *f* is bounded and of class $\mathcal {C}^{1}$. Moreover, *f* can be written as $f(s)=sg(s)$ with $g:[0,+\infty )\longrightarrow (0,+\infty )$ strictly decreasing.

### Mathematical analysis of model (4.2)

First, we prove some basic properties of boundedness and permanence for the sequences of (4.2).

#### Proposition 4.1

There exist two positive constants $K_{1}$ and $K_{2}$ so that

$$\begin{aligned} \limsup _{n\rightarrow +\infty } J_{n}\le K_{1}\;\;\;and\;\;\;\limsup _{n\rightarrow +\infty } A_{n}\le K_{2} \end{aligned}$$

for all $$(J_{0},A_{0})\in [0,+\infty )^2$$.

#### Proof

Let *M* be an upper bound of *f*. Consider $K_{2}=\frac{M}{1-\mu }$ and $K_{1}=ak K_{2}+\frac{\gamma k}{\eta }$. Take $(J_{n},A_{n})$ a sequence from (4.2) with initial condition $(J_{0},A_{0})\in [0,+\infty )^2$. From the second equation in (4.2), we deduce that

$$\begin{aligned} A_{n+1}\le M+\mu A_{n}. \end{aligned}$$

This implies that

$$\begin{aligned} A_{n+1}\le \frac{M(1-\mu ^{n+1})}{1-\mu }+\mu ^{n+1}A_{0} \end{aligned}$$

for all $n\in \mathbb {N}$. Since $\mu \in (0,1)$, we deduce that $\limsup _{n\rightarrow +\infty }A_{n}\le K_{2}$. From the first equation in (4.2), we have that

$$\begin{aligned} J_{n+1}\le a k A_{n}+\frac{\gamma k}{\eta }. \end{aligned}$$

This implies that $\limsup _{n\rightarrow +\infty } J_{n}\le K_{1}$. $\square$

Let $H:[0,+\infty )^2\longrightarrow [0,+\infty )^2$ be the map associated with model (4.2), that is,

$$\begin{aligned} H(J,A)=\left( \frac{ak A}{1+\eta J}+\frac{\gamma k J}{1+\eta J}, f(J)+\mu A \right) . \end{aligned}$$

We say that the origin is locally unstable if there is $r>0$ with the following property: For each $(J_{0},A_{0})\in (B((0,0),r)\backslash \{(0,0)\})\cap [0,+\infty )^2$, there is $m=m(J_{0},A_{0})\in \mathbb {N}$ so that

4.3 $$\begin{aligned} H^{m}(J_{0},A_{0})\not \in {\overline{B}}((0,0),r) \end{aligned}$$

where *B*((0, 0), *r*) and ${\overline{B}}((0,0),r)$ denote the open and closed balls with center (0, 0) and radius *r*, respectively. It is well known that if $\rho (J_{H}(0,0))>1$, then the origin is locally unstable where $\rho (J_{H}(0,0))$ denotes the spectral radius of the Jacobian matrix of *H* at (0, 0).

#### Theorem 4.1

If the origin is locally unstable, then there is $\delta >0$ so that

4.4 $$\begin{aligned} \liminf _{n\rightarrow +\infty } J_{n}\ge \delta \;\;\;and\;\;\;\liminf _{n\rightarrow +\infty } A_{n}\ge \delta \end{aligned}$$

for all $(J_{0},A_{0})\in (0,+\infty )^2$. Moreover, there exists an equilibrium $(J_{*},A_{*})\in (0,+\infty )^2$ of model (4.2).

#### Proof

Since (0, 0) is locally unstable, there is $r>0$ so that the ball *B*((0, 0), *r*) satisfies (4.3). Define $\mathcal {R}=[0,K_{1}+1]\times [0,K_{2}+1]$ with $K_{1}$ and $K_{2}$ the positive constants given in Proposition 4.1. We know that, any orbit of (4.2) enters into $\mathcal {R}$ and remains inside after a certain number of iterations. Take

$$\begin{aligned} \Delta =\mathcal {R}\backslash B((0,0),r). \end{aligned}$$

Using that $H([0,+\infty )^2\backslash \{(0,0)\})\subset (0,+\infty )^2$ and property (4.3), for each $(J_{0},A_{0})\in \Delta$, there is $n=n(J_{0},A_{0})\in \mathbb {N}$ and $\epsilon =\epsilon (J_{0},A_{0})>0$ so that

$$\begin{aligned} H^{n}(B((J_{0},A_{0}),\epsilon )\cap \Delta )\subset Int \Delta . \end{aligned}$$

Since $\Delta$ is a compact set, we can find a finite family of points $p_{1},...,p_{t}\in \Delta$, a finite family of natural numbers $n_{1},...,n_{t}\in \mathbb {N}$ and a finite family of positive numbers $\epsilon _{1},....,\epsilon _{t}$ with the following properties:

1. $$\Delta \subset \bigcup _{j=1}^{t} B(p_{j},\epsilon _{j})\cap \Delta $$.
2. $$H^{n_{j}}(B(p_{j},\epsilon _{j})\cap \Delta )\subset Int \Delta $$.

Take $n^*=\max \{n_{1},...,n_{t}\}$. Let us prove that $\Gamma =\Delta \cup H(\Delta )\cup ....\cup H^{n^*-1}(\Delta )$ is positively invariant, i.e., $H(\Gamma )\subset \Gamma$. To see this claim, it is enough to prove that $H^{n^*}(\Delta )\subset \Gamma$. Given $q\in \Delta$, there is an index $j_{0}$ so that $q\in B(p_{j_{0}},\epsilon _{j_{0}})\cap \Delta$ by **(P1)**. Thus, using **(P2)**,

$$\begin{aligned} H^{n^*}(q)=H^{n^*-n_{j_{0}}}\left( H^{n_{j_{0}}}(q)\right) \subset H^{n^*-n_{j_{0}}}\left( Int\Delta \right) . \end{aligned}$$

Collecting all the information, we have that given an orbit $\{(J_{n},A_{n})\}$ with $(J_{0},A_{0})\in [0,+\infty )^2\backslash \{(0,0)\}$, the sequence enters into $\Gamma \subset [0,+\infty )^2\backslash \{(0,0)\}$. Using that $\Gamma$ is a compact set and $H(\Gamma )\subset (0,+\infty )^2$, we can find $\delta >0$ so that $H(\Gamma )\subset [\delta ,+\infty )^2$. Now, the proof of (4.4) is completed. The existence of the equilibrium $(J_{*},A_{*})\in (0,+\infty )^2$ is well-known when the system is permanent (see Hutson 1990; Smith and Thieme 2011). $\square$

Throughout the rest of the section, we assume that an equilibrium $(J_{*},A_{*})\in (0,+\infty )^2$ exists. Note that $(J_{*},A_{*})$ satisfies

4.5 $$\begin{aligned} \left\{ \begin{array}{lll} 1=\frac{ k}{1+\eta J_{*}}\left( a\frac{A_{*}}{J_{*}} +\gamma \right) \\[2ex] A_{*}=\frac{f(J_{*})}{1-\mu }. \end{array}\right. \end{aligned}$$

Next, we employ the change of variable

$$\begin{aligned} x_{n}=\frac{J_{n}}{J_{*}}\;\;\;\textrm{and}\;\;\; y_{n}=\frac{A_{n}}{A_{*}}. \end{aligned}$$

After some straightforward computations, we arrive at

4.6 $$\begin{aligned} \left\{ \begin{array}{lll} x_{n+1}=\frac{k}{1+\eta J_{*} x_{n}}\left( a\frac{A_{*}}{J_{*}}y_{n}+\gamma x_{n}\right) \\[2ex] y_{n+1}=(1-\mu )\frac{f(J_{*}x_{n})}{f(J_{*})}+\mu y_{n}. \end{array}\right. \end{aligned}$$

We have used the second equation of (4.5) in the second equation of (4.6). Now, we are ready to give the main result of this section.

#### Theorem 4.2

Assume that the origin is locally unstable in (4.2) and $f:[0,+\infty )\rightarrow [0,+\infty )$ is strictly increasing. For any sequence $\{(J_{n},A_{n})\}$ of model (4.2) with initial condition $(J_{0},A_{0})\in (0,+\infty )^2$,

$$\begin{aligned} \lim _{n\rightarrow +\infty }(J_{n},A_{n})=(J_{*},A_{*}). \end{aligned}$$

To prove this theorem, we need several preliminary results.

#### Lemma 4.1

Assume that the origin is locally unstable in (4.2). Then, the following inequalities hold:

(i) $$\frac{f(J_{*}x)}{f(J_{*})}>x$$ for all $$x\in (0,1)$$.
(ii) $\frac{f(J_{*}x)}{f(J_{*})}<x$ for all $x\in (1,+\infty )$.

#### Proof

$\frac{f(J_{*}x)}{f(J_{*})}>x$ for all $x\in (0,1)$ is equivalent to $J_{*}x g(J_{*}x)>J_{*}x g(J_{*})$. This last inequality is obviously true because *g* is strictly decreasing. The proof of **(ii)** is analogous. In this lemma, we have imposed that the origin is locally unstable to have a nontrivial equilibrium $(J_{*},A_{*})$. $\square$

#### Lemma 4.2

Assume that the origin is locally unstable in (4.2) and $f:[0,+\infty )\rightarrow [0,+\infty )$ is strictly increasing. Given a sequence $\{(x_{n},y_{n})\}$ of (4.6) with initial condition $(x_{0},y_{0})\in (0,+\infty )^2$, we define

4.7 $$\begin{aligned} \left\{ \begin{array}{lll} L=\min \{\liminf _{n\rightarrow +\infty }x_{n},\liminf _{n\rightarrow +\infty }y_{n}\}\\[2ex] S=\max \{\limsup _{n\rightarrow +\infty }x_{n},\limsup _{n\rightarrow +\infty }y_{n}\}. \end{array}\right. \end{aligned}$$

Then, we have the following:

(i) $$L\le 1$$ and $$S\ge 1$$.
(ii) $L=1$ and $S>1$ can not occur.
(iii) $S=1$ and $L<1$ can not occur.

#### Proof

First, we observe that by Proposition 4.1 and Theorem 4.1, *L* and *S* are well defined and $L,S\in (0,+\infty )$. Now, we focus on proving that $L\le 1$. Assume, by contradiction, that $L>1$. We distinguish between two cases:

**Case 1:** $S=\limsup _{n\rightarrow +\infty } x_{n}$.Take $\sigma (n)$ a subsequence so that $\lim _{n\rightarrow +\infty }x_{\sigma (n)+1}= S$. Using that $\{x_{n}\}$ and $\{y_{n}\}$ are bounded, it is not restrictive to assume that $\lim _{n\rightarrow +\infty }y_{\sigma (n)}=S_{1}$ and $\lim _{n\rightarrow +\infty }x_{\sigma (n)}= S_{2}$ with $S_{1},S_{2}\in [L,S]$. Evaluating the first equation of (4.6) at $\sigma (n)$ and making $n\rightarrow +\infty$, we arrive at

4.8 $$\begin{aligned} S=\frac{k}{1+\eta J_{*}S_{2}}\left( a \frac{A_{*}}{J_{*}} S_{1}+\gamma S_{2}\right) . \end{aligned}$$

Using that $S_{1},S_{2}\le S$, we deduce that

4.9 $$\begin{aligned} 1\le \frac{k}{1+\eta J_{*} S_{2}}\left( a\frac{A_{*}}{J_{*}}+\gamma \right) . \end{aligned}$$

Since $L>1$ and $S_{2}\ge L$, we have that $S_{2}>1$. Inserting this inequality in (4.9), we obtain a contradiction with the first equation of (4.5).

**Case 2:** $S=\limsup _{n\rightarrow +\infty } y_{n}$.Arguing as in **Case 1**, using the second equation of (4.6), we find two positive constants $S_{1},S_{2}\in [L,S]$ with

4.10 $$\begin{aligned} S=\frac{(1-\mu )f(J_{*}S_{1})}{f(J_{*})}+\mu S_{2}. \end{aligned}$$

This implies that

4.11 $$\begin{aligned} S\le \frac{f(J_{*}S_{1})}{f(J_{*})} \end{aligned}$$

since $S_{2}\le S$. On the other hand, using that $L>1$ and $S_{1}\ge L$, we have $S_{1}>1$. Then, by Lemma 4.1,

4.12 $$\begin{aligned} \frac{f(J_{*}S_{1})}{f(J_{*})}<S_{1}\le S. \end{aligned}$$

Obviously, (4.11) and (4.12) are contradictory. After this argument, we have proved that $L\le 1$. To prove that $S\ge 1$, we have to employ a similar argument.Next, we focus on the proof of **(ii)**. Assume, by contradiction, that $L=1$ and $S>1$ hold. We follow the same strategy as in the proof of **(i)**. If $S_{2}>1$ in **Case 1**, we have already found a contradiction in (4.9), (with the same argument as before). If $S_{2}=1$ in **Case 1**, we achieve from (4.8) that

$$\begin{aligned} S=\frac{k}{1+\eta J_{*}}\left( a\frac{A_{*}}{J_{*}}S_{1}+\gamma \right) . \end{aligned}$$

Using that $S_{1}\le S$ and $1<S$, we deduce that

$$\begin{aligned} 1<\frac{k}{1+\eta J_{*}}\left( a\frac{A_{*}}{J_{*}}+\gamma \right) , \end{aligned}$$

a contradiction with the first equation of (4.5). On the other hand, if $S_{1}>1$ in **Case 2**, the proof is the same as before. If $S_{1}=1$ in **Case 2**, (4.11) reads as $S\le 1$, a contradiction. The proof of **(iii)** would be analogous using the argument to prove $S>1$. $\square$

#### Lemma 4.3

Assume that the origin is locally unstable in (4.2) and $f:[0,+\infty )\longrightarrow [0,+\infty )$ is strictly increasing. Take $\{(x_{n},y_{n})\}$ a sequence from (4.6) with initial condition $(x_{0},y_{0})\in (0,+\infty )^2$. With the notation of Lemma 4.2, if $L<1<S$, then

$$\begin{aligned} L=\liminf _{n\rightarrow +\infty }x_{n}\;\;\;and\;\;\;S=\limsup _{n\rightarrow +\infty }x_{n}. \end{aligned}$$

#### Proof

Assume, for instance, that $L=\liminf _{n\rightarrow +\infty }y_{n}$. Let us look for a contradiction. Take $\sigma (n)$ a subsequence so that $\lim _{n\rightarrow +\infty }y_{\sigma (n)+1}= L$. It is not restrictive to suppose that $\lim _{n\rightarrow +\infty }y_{\sigma (n)}= L_{1}$ and $\lim _{n\rightarrow +\infty }x_{\sigma (n)}= L_{2}$ with $L<1$ and $L_{1},L_{2}\ge L$. Evaluating the second equation of (4.6) at $\sigma (n)$ and making $n\rightarrow +\infty$, we arrive at

$$\begin{aligned} L=\frac{(1-\mu )f(J_{*}L_{1})}{f(J_{*})}+\mu L_{2}. \end{aligned}$$

Since $L_{2}\ge L$, we have that

4.13 $$\begin{aligned} L\ge \frac{f(J_{*}L_{1})}{f(J_{*})}. \end{aligned}$$

On the other hand, $L\le L_{1}$ and *f* strictly increasing imply that

$$\begin{aligned} \frac{f(J_{*}L)}{f(J_{*})}\le \frac{f(J_{*}L_{1})}{f(J_{*})}. \end{aligned}$$

Moreover, since $$L<1$$,

$$\begin{aligned} L<\frac{f(J_{*}L)}{f(J_{*})}, \end{aligned}$$

by Lemma 4.1. This last inequality contradicts (4.13). To prove that $S=\limsup _{n\rightarrow +\infty }x_{n}$, we have to reason analogously. $\square$

Consider $\Psi (x,y)=\frac{k}{1+\eta J_{*}x}\left( a\frac{A_{*}}{J_{*}}y+\gamma x\right)$. After a simple computation, we deduce the following:

**(Q1)**: If $$y<\frac{\gamma }{\eta a A_{*}}$$, $$\frac{\partial \Psi }{\partial x}(x,y)>0$$.
**(Q2)**: If $$y>\frac{\gamma }{\eta a A_{*}}$$, $$\frac{\partial \Psi }{\partial x}(x,y)<0$$.

#### Proof of Theorem 4.2

Take $\{(J_{n},A_{n})\}$ a sequence obtained from (4.2) with initial condition $(J_{0},A_{0})\in (0,+\infty )^2$. We have to prove that $\lim _{n\rightarrow +\infty }J_{n}=J_{*}$ and $\lim _{n\rightarrow +\infty }A_{n}= A_{*}$, or equivalently, $\lim _{n\rightarrow +\infty }x_{n}=\lim _{n\rightarrow +\infty }\frac{J_{n}}{J_{*}}= 1$ and $\lim _{n\rightarrow +\infty }y_{n}=\lim _{n\rightarrow +\infty }\frac{A_{n}}{A_{*}}= 1$. Using the notation of Lemma 4.2, it is enough to prove that

4.14 $$\begin{aligned} L=S=1. \end{aligned}$$

Assume, by contradiction, that (4.14) does not hold. Then, $L\not =S$. Note that, by Lemma 4.2 (i), $L=S=\xi$ with $\xi \not =1$ can not occur. Moreover, by Lemma 4.2 (ii) (resp. (iii)), the case $L=1$ and $S>1$ (resp. $L<1$ and $S=1$) cannot occur either. Thus, it is not restrictive to assume that $L<1<S$, otherwise the proof is completed. By Lemma 4.3, we know that $L=\liminf _{n\rightarrow +\infty }x_{n}$ and $S=\limsup _{n\rightarrow +\infty }x_{n}$. We focus on *L*. We take $\sigma (n)$ a subsequence so that $\lim _{n\rightarrow +\infty }x_{\sigma (n)+1}= L.$ It is not restrictive to suppose that $\lim _{n\rightarrow +\infty }x_{\sigma (n)}= L_{1}$ and $\lim _{n\rightarrow +\infty }y_{\sigma (n)}= L_{2}$ with $L<1$ and $L_{1},L_{2}\ge L$. Evaluating at $\sigma (n)$ the first equation of (4.6) and making $n\rightarrow +\infty$, we arrive at

4.15 $$\begin{aligned} L=\frac{k}{1+\eta J_{*}L_{1}}\left( a\frac{A_{*}}{J_{*}}L_{2}+\gamma L_{1}\right) . \end{aligned}$$

If $L_{2}<\frac{\gamma }{\eta a A_{*}}$, by **(Q1)** together with $L\le L_{1}$, we obtain that

$$\begin{aligned} L\ge \frac{k}{1+\eta J_{*}L}\left( a\frac{A_{*}}{J_{*}}L_{2}+\gamma L\right) . \end{aligned}$$

Using that $L\le L_{2}$, the previous inequality implies that

$$\begin{aligned} 1\ge \frac{k}{1+\eta J_{*}L}\left( a\frac{A_{*}}{J_{*}}+\gamma \right) , \end{aligned}$$

a contradiction with the first equation of (4.5) since $L<1$. If $L_{2}\ge \frac{\gamma }{\eta a A_{*}}$, we obtain from (4.15) that

4.16 $$\begin{aligned} L\ge \frac{k\gamma }{1+\eta J_{*} L_{1}}\left( \frac{1}{\eta J_{*}}+L_{1}\right) =\frac{k\gamma }{\eta J_{*}}. \end{aligned}$$

Working with *S* analogously, we deduce that

4.17 $$\begin{aligned} S\le \frac{k\gamma }{\eta J_{*}}. \end{aligned}$$

By (4.16) and (4.17), we conclude that $S=L=\frac{k\gamma }{\eta J_{*}}$, a contradiction. $\square$

## Discussion

A major challenge in theoretical ecology is understanding the mechanisms that produce population oscillations. Despite many advances in the field, many important questions remain to be solved. One tenet that resulted from early investigations of differential equations is that time delays can create oscillations (Gurney et al. 1980). For example, this piece of biological folklore is behind the oscillations observed in some vole populations in nature (Hanski et al. 1993). However, time delays are sometimes harmless (El-Morshedy and Gopalsamy 2003; El-Morshedy and Ruiz-Herrera 2024). Describing the role of time delays in particular situations is normally a hard task. This paper analyzes this role through the classical Clark’s model, a simple and versatile model in ecology (Brauer and Castillo-Chavez 2012; Clark 1976; Milton and Bélair 1990; Thieme 2018; Yu and Li 2022). We have discussed the dynamical differences between populations where the main intraspecific competition episodes occur during the reproduction period or during a different one. The main conclusion is that the second situation is more prone to present population oscillations. Ticks typically fall into the second situation and our results are quite consistent with previous analyses conducted with other modeling frameworks (El-Morshedy and Ruiz-Herrera 2024; Huang et al. 2022; Zhang and Wu 2020). On the other hand, a common phenomenon of both situations is that increasing $\mu$ in (3.24) or (3.27) suppresses population oscillations. In other words, increasing the density-independent surviving probability of adults produces simple dynamics in the model, and time delays are harmless. To deduce these biological insights, we have proposed an extension of the classical “decomposing+embedding” method in Enciso et al. (2006), Kulenović and Merino (2006), Smith (2006, 2008). Our contributions in comparison with those papers are i) to relax some of the usual conditions required in the original method, and ii) to provide more models in which this type of argument works.

Another goal of the paper was to study the influence of the stage structure on the dynamical behavior of a biological population through (4.2). This model was proposed originally to study the dynamical behavior of wild mosquitoes (Yu and Li 2022). However, it does not contain any specific aspects/characteristics of mosquitoes that affect the model except for the maturation delay reflecting the age structure, a common trait of many species. The model is rather simple, only involving survival and recruitment terms in each group. Our results suggested that adult recruitment plays an important role in the population dynamics. Specifically, recruitments associated with intraspecific competitions of contest type (Brannstrom and Sumpter 2005) do not produce sustainable oscillations in the long term. From a mathematical point of view, we have offered a novel strategy to handle planar systems based on subtle properties of scalar discrete equations. As we will see in forthcoming papers, this approach is useful to analyze other biological situations. Moreover, this paper concludes that the conditions of Theorem 3.1 in Yu and Li (2022) also imply global attraction.

As mentioned in Sect. 2, the classical Clark model with $k=1$ can be derived from (2.1) when the population is semelparous ($\mu _{2}=0$) or there is a rapid development to maturity ($p=1$). Specifically,

5.1 $$\begin{aligned} \left\{ \begin{array}{lll} x_{n+1}=\mu _{1}(1-p)x_{n}+f(y_{n})\\[2ex] y_{n+1}=\mu _{1} p x_{n}+\mu _{2} y_{n} \end{array}\right. \end{aligned}$$

leads to

5.2 $$\begin{aligned} y_{n+1}=\mu _{2}y_{n}+\mu _{1}f(y_{n-1}) \end{aligned}$$

for $$p=1$$ and

**Fig. 4:**
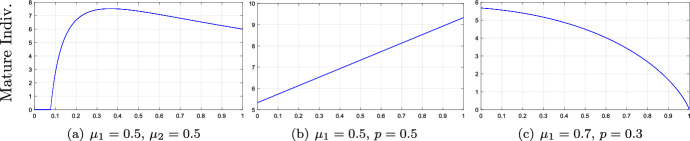
Bifurcation diagrams of model (5.1) with $f(x)=7 x/(1+x)$ ((a)-(b)) and $f(x)=8 x e^{-x}$ (c). Bifurcation parameters (a) $p\in [0,1]$; (b)-(c) $\mu _{2}\in [0,1]$

5.3 $$\begin{aligned} y_{n+1}=\mu _{1}(1-p)y_{n}+\mu _{1}pf(y_{n-1}) \end{aligned}$$

for $\mu _{2}=0$. A natural question is to establish connections between (5.2) or (5.3) and (5.1). In biological terms, we describe the role of the reproduction strategy and the developmental rate on the population dynamics. We normally obtain a unimodal response concerning the population density of mature individuals by varying the developmental rate *p* in model (5.1). Small values of *p* imply a reduced density of mature population and so, the global extinction of the whole population. Note that only the mature population contributes to reproduction, (Fig. 4a). On the other hand, we can expect any response in (5.1) by varying $\mu _{2}$, (Fig. 4 b-c).

**Fig. 5:**
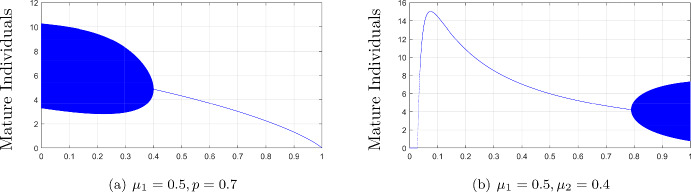
Bifurcation diagrams of model (5.1) with $f(x)=25 xe^{-x}$. Bifurcation parameters (a) $\mu _{2}\in [0,1]$ (b) $p\in [0,1]$. Model (5.1) exhibits the most complex dynamical behaviors when Clark’s model is obtained

Regarding the dynamical behavior of model (5.1) compared to (5.2) and/or (5.3), the main conclusion is that Clark’s model typically displays more complex behaviors than (5.1), (Fig. 5). Reducing *p* and/or increasing $\mu _{2}$ are mechanisms that increase the population’s mortality rate. In agreement with previous results in the literature, (see Liz and Ruiz-Herrera (2012b) and the references therein), these mechanisms play a stabilizing role.
